# Stereotactic Radiosurgery for Patients with Brain Metastases from Sarcomas

**DOI:** 10.3390/cancers17132118

**Published:** 2025-06-24

**Authors:** Andrew Hoang, Zhishuo Wei, Constantinos G. Hadjipanayis, Ajay Niranjan, L. Dade Lunsford

**Affiliations:** 1 School of Medicine, University of Pittsburgh, Pittsburgh, PA 15213, USA; 2 Center for Image-Guided Neurosurgery, University of Pittsburgh Medical Center, Pittsburgh, PA 15213, USA; 3 Department of Neurological Surgery, University of Pittsburgh Medical Center, Pennsylvania, PA 15213, USA

**Keywords:** brain metastases, stereotactic radiosurgery, sarcoma, tumor control

## Abstract

Purpose: We present our single-institution experience of sarcomatous brain metastasis patients who underwent stereotactic radiosurgery (SRS) over the past 35 years. Methods: In total, 31 patients (16 males) who underwent SRS for sarcoma brain metastases were identified. Median age at presentation to SRS was 47 (range: 4–78) months. Common histopathologies included leiomyosarcoma (eight patients), osteosarcoma (six patients), alveolar sarcoma (three patients), Ewing sarcoma (three patients), and undifferentiated/unclassified sarcoma (three patients). The median Karnofsky Performance Score (KPS) was 90. Nine patients underwent pre-SRS craniotomy. The median dose prescribed was 18 Gy. The median cumulative tumor volume was 1.4 cc. Results: Median patient overall survival (OS) after SRS was 7 (range: 0–155) months. Local tumor control (LTC) was achieved in 105 out of 113 tumors, at a median time of 3 (range: 0–17) months between SRS and progression. LTC rates per patient and per tumor were 74.2% and 92.9%, respectively. Following SRS, 10 patients (32.3%) developed new tumors at a median time of 6 (range: 1–25) months. Four patients experienced adverse radiation effects (AREs). At the last follow-up, all patients died, one patient from intracranial progression, 27 from systemic disease progression, and the remaining from unrelated medical conditions. Conclusions: Given high LTC and low ARE rates, this suggests SRS as a strong candidate for the non-invasive management of sarcomatous brain metastases, which typically present late following initial presentation of the primary disease.

## Simple Summary

Sarcomatous brain metastases are exceedingly rare and generally associated with a poor prognosis, resulting in limited life expectancy for affected patients. There are no current standard treatment guidelines for these malignancies. Stereotactic radiosurgery (SRS) offers a targeted, non-invasive approach for brain metastases. In this retrospective study, we evaluated the clinical outcomes of patients with sarcoma brain metastases who underwent SRS to determine and optimize SRS treatment parameter settings. LTC rates per patient and per tumor were 74.2% and 92.9%, respectively. The occurrence of new, untreated brain metastases was managed with repeat SRS. The results of this study suggest SRS as a safe, efficient modality for the management of sarcomatous brain metastases and improving patient longevity.

## 1. Introduction

Sarcomatous brain metastases represent a rare manifestation of the primary cancer, reporting an incidence rate of ~1–8% [1,2]. Prognosis is generally poor among affected patients, with survival after brain metastasis diagnosis ranging between 2 and 7 months [3]. However, improvements in systemic management approaches have prolonged longevity of life and ensured the better detection of these brain metastases [4]. No standardized guidelines currently exist for managing patients with brain metastases originating from sarcomas [1]. Management options include surgical resection [5], SRS [6], whole-brain radiation therapy (WBRT) [7], and systemic therapy (cytotoxic chemotherapy, immunotherapy, molecularly targeted therapy) [8,9].

Gross total resection is generally indicated for larger tumors in patients with controlled systemic disease [10,11] but faces challenges addressing tumors with multiple brain metastases [12]. WBRT is a minimally invasive approach that aims to address residual metastases and those with leptomeningeal spread but is highly associated with neurocognitive defects [13,14]. Systemic therapy strategies also provide an alternative for patients seeking minimally invasive metastatic tumor management. However, the blood–brain barrier (BBB) limits the entry of most therapeutic agents from reaching the locations of tumors [15,16]. Immunotherapy has shown potential benefit in overcoming the BBB but is limited in its effectiveness by the tumor microenvironment [17,18].

Stereotactic radiosurgery (SRS) is a non-invasive primary or adjuvant strategy that is generally considered for more common cancers that metastasize to the brain. Previous studies report that 1-year local tumor control rates of brain metastases derived from general primary cancers are >85% [19,20]. Here, we report the outcomes of patients with primary sarcomas that metastasized to the brain who underwent SRS.

## 2. Materials and Methods

### 2.1. Patient Characteristics

We performed a retrospective analysis of the Center for Image-Guided Neurosurgery database at the University of Pittsburgh Medical Center between 1987 and 2024. Individual electronic health records and radiographic imaging studies were reviewed to gather data on patient management and clinical outcomes. Patients with primary sarcomas and metastatic disease to the brain confirmed by radiographic imaging studies (via computed tomography and/or magnetic resonance imaging [MRI]) were included in this study.

### 2.2. SRS Technique

Technical aspects of Gamma Knife SRS procedures have been described extensively in our previous studies [21]. We briefly describe the procedure here. Placement of the Leksell stereotactic headframe was performed following the administration of mild intravenous sedation and local anesthesia. The gadolinium contrast agent was administered prior to the T-1-weighted brain MRI sequence with 1.5 mm axial slices. Axial fast spin echo T2-weighted images of the whole brain were obtained at a 3 mm slice thickness. Tumor volumes were delineated by color-coded markers as a guide for dose planning. Various models of the Leksell Gamma Knife units were used in this study, including models B, C, 4C, Perfexion, and ICON. Patients were discharged the same day following SRS. The prescription maximal and margin doses for targeted tumors were based upon the characteristics of the tumor, including its volume, primary histopathology, location relative to critical structures, and any previous exposure to the brain (radiation approach). Smaller lesions situated further from critical structures received higher margin doses.

### 2.3. Patient Follow-Up

MRI studies were performed at 3-month intervals during the first year following SRS and increased to 6 months after if no new brain lesions were detected. At each clinical follow-up, high-definition MRIs with and without contrast were obtained to assess tumor response to SRS, according to the Response Assessment in Neuro-Oncology Brain Metastases (RANO-BM) criteria [22]. Local tumor progression was defined as an increase in tumor size exceeding 20%, while complete response was characterized by a reduction in size greater than 50%. Any tumors that did not fulfill the criteria denoted above were classified as stable disease. New brain metastases were defined as the emergence of new tumor lesions in areas not previously managed with SRS. Adverse radiation effects (AREs) were defined as the development of increased peri-tumoral edema or ARE with pathology confirmation.

### 2.4. Study Endpoints

The primary endpoint of this study was local tumor control (LTC), which was calculated from the date of SRS to the date of tumor progression or the date of the last radiographic imaging follow-up. Notable secondary endpoints included overall survival (OS), which was defined as the duration between the date of SRS and the date of the last clinical follow-up or date of death. The new brain metastasis-free rate represented the duration between the date of SRS and the date of radiologically confirmed presence of a new or untreated tumor or last imaging follow-up.

### 2.5. Statistical Analysis

Microsoft Excel^®^ Version 2024 (Washington, DC, USA) was used to record and analyze patient, SRS, and outcome characteristics. OS curve, LTC curve, and new brain metastasis-free rate curve statistical analyses were generated using Prism Version 10.3.1 (GraphPad, San Diego, CA, USA). Kaplan–Meier was used for univariate analysis. Patients with local tumor progression and last clinical/imaging study follow-up were included in the analysis of LTC and new brain metastasis-free rate curves. Information recorded in the tables was represented as mean ± standard deviation (median [range]), with all numerical values rounded to the nearest hundredth.

## 3. Results

### 3.1. Patient and SRS Management Characteristics

Patient demographics and radiographic imaging characteristics are outlined in Table 1. All data collection followed the guidelines outlined by the institutional review board. Thirty-one patients (16 males) presented with brain metastases from primary sarcomas. The median age of the primary disease diagnosis was 44 (range: 3–77) years. The median time from primary diagnosis of sarcoma to brain metastasis was 20 (range: 0–183) months. The median age at SRS was 47 (range: 4–78) years. Active systemic disease was reported in 27 patients. The median KPS at SRS was 90 (range: 40–100). The histology included leiomyosarcoma (eight patients), osteosarcoma (six patients), alveolar sarcoma (three patients), Ewing sarcoma (three patients), undifferentiated/unclassified sarcoma (three patients), chondrosarcoma (two patients), other (three patients), pleomorphic sarcoma (two patients), liposarcoma (one patient), rhabdomyosarcoma (one patient), and synovial sarcoma (one patient). Prior to SRS, seven patients failed initial WBRT. Concurrent systemic disease management included cytotoxic chemotherapy (four patients) and targeted therapy (one patient).

The SRS procedure parameters are documented in Table 2. One hundred and thirteen total brain metastases underwent SRS and the median number of brain tumors per patient was two tumors (range: 1–9). The median margin dose was 18 Gy (range: 10–20 Gy) and the median maximal dose was 29.45 Gy (range: 19.2–44.4 Gy). The median isodose was 50% (range: 40–85%). The median cumulative tumor volume was 1.4 cc (range: 0.0041–38.4 cc). The median cumulative 12 Gy volume was 9.85 cc (range: 0.0187–40). All patients underwent single-session SRS.

### 3.2. Overall Survival

Patient outcomes are described in Table 3. The median survival after SRS was 7 (range: 0–155) months. At the last clinical follow-up, all patients were deceased at follow-up. Twenty-seven patient deaths were related to systemic disease progression. The one-year survival rate after the diagnosis of their brain metastasis was 54.8%. The 6-, 12-, and 24-month survival rates from SRS were 51.6%, 45.2%, and 29.0%, respectively. On univariate analysis (Table 4), patients with extracranial disease were significantly associated with worse overall survival outcomes.

### 3.3. Tumor Control

Eight patients exhibited local tumor progression following initial SRS but underwent repeat SRS thereafter. The median interval between the date of initial SRS and radiologically confirmed tumor progression was 3 (range: 0–17) months. Local tumor progression only occurred in eight cases out of 113 tumors recorded. LTC per tumor was 92.9%. The most common pathologies for patients with tumors that progressed were osteosarcoma (11 tumors), leiomyosarcoma (8 tumors), undifferentiated sarcoma (4 tumors), and pleomorphic sarcoma (2 tumors). Given the small patient size, univariate analysis did not clearly exhibit any associations with LTC (Table 5).

**Figure 1:**
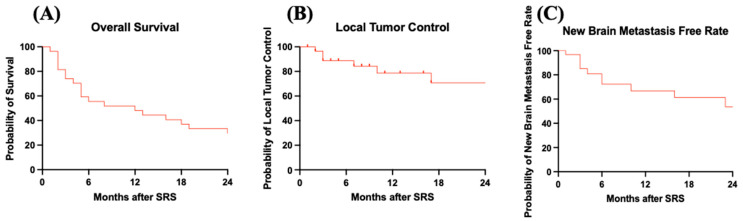
Kaplan–Meier curves for patients who received SRS for sarcoma brain metastasis management. (**A**) OS curve: median overall survival was 7 months. (**B**) LTC curve: overall LTC was 74.19%. (**C**) New brain metastasis-free rate curve: overall new brain metastasis-free rate was 67.74%.

Ten patients developed additional tumors during the observation interval. The median time between initial SRS and new tumor development was 6 (range: 1–25) months. Six patients again underwent SRS. The 12-month new brain metastasis-free rate was 74.19%, as shown in Figure 1. The most common pathologies for the patients with new tumor development were undifferentiated sarcoma (six tumors), osteosarcoma (three tumors), Ewing sarcoma (three tumors), pleomorphic sarcoma (one tumor), leiomyosarcoma (two tumors), and rhabdomyosarcoma (one tumor).

### 3.4. Adverse Radiation Effects

Four patients exhibited asymptomatic peritumoral reactive edema via follow-up radiography as a manifestation of AREs following SRS. Of these patients, one patient exhibited imaging changes compatible with radiation necrosis. The median time of the development of ARE was 2.50 (range: 0–5.0) months. Two patients required additional surgical resection due to progressive edema.

## 4. Discussion

In this retrospective, single-institutional study, we evaluated data collected from our series of 31 patients with sarcoma brain metastases who underwent SRS.

### 4.1. Epidemiology and Natural History of Sarcoma Brain Metastases

Sarcoma brain metastases are extremely rare compared to lung, renal, and breast cancers [4], with an incidence ranging from 1 to 8% [4,6]. The onset of metastatic disease typically occurs late in the course of primary disease progression, with an interval from primary cancer to brain metastasis diagnosis of less than 2 years [23,24]. Common primary histopathology of sarcoma types includes leiomyosarcoma, undifferentiated pleomorphic sarcoma, and fibrosarcoma [4,23,24]. Due to the rarity of brain metastasis from sarcoma, the published literature on the outcomes is sparse. The reported median interval between diagnoses ranged from 12 to 30 months, indicating a late progression of the primary disease [3,4,23,25,26,27,28,29]. Kokkali et al. reported that the median time from initial sarcoma diagnosis to brain metastasis diagnosis was 16 months among a retrospective cohort study of 34 patients [24]. The present series reported the median time between primary sarcoma and brain metastasis diagnosis to be 20 (range: 0–183) months.

### 4.2. Fractionated Whole-Brain Radiation Therapy (WBTR) and Surgery

Prior to the inception of SRS, primary WBRT or surgical resection (followed by WBRT) was performed to manage brain metastases. Indications for surgical resection were primarily recommended for large tumors with mass effect [30], need for histologic diagnosis [30], controlled systemic disease [5], good performance status (KPS > 60) [5,26], and certain histological subtypes [5,31]. The current literature reported a survival interval range of 7–25 months for patients following surgical resection alone [5,26,32,33]. Palliative WBRT is usually indicated for patients with diffuse brain metastases and/or poor performance status (KPS < 70) [30,34,35]. Zhang et al. reported a median OS of 8.3 months after undergoing WBRT management alone for sarcoma-specific brain metastases [23]. Kokkali et al. reported a median OS of 3 months after undergoing WBRT management alone for sarcoma-specific brain metastases [24]. The literature is sparse for the combined use of WBRT and surgical resection for sarcoma-specific brain metastases, but Vecht et al. and Patchell et al. reported median OS of 10 months and 9.2 months for general brain metastasis management [36,37].

### 4.3. Indication of SRS for Sarcomas

Over the last few decades, SRS has emerged as a minimally invasive management option for patients with brain metastasis refractory to surgical resection or WBRT. SRS is indicated for patients who do not meet the eligibility requirements for surgery [38]. Unlike WBRT, SRS does not directly interfere with ongoing cycles of systemic therapy and minimizes the risk of neurocognitive sequelae for management of the primary cancer [39]. Previous studies have reported clinical outcomes of sarcoma brain metastases managed via SRS as the primary modality [40,41]; these are provided as a summary in Table 6.

Flannery et al. reported a median OS of 5 months in 21 patients with sarcoma brain metastases after SRS management [42]. Zamarud et al. reported a median OS of 8.2 months for patients with sarcoma brain metastases who underwent SRS management [6]. Chaigneau et al. reported a median OS of 10.2 months for patients with sarcoma brain metastases who underwent SRS management [4]. In the present study, we reported a median OS of 7 months for 31 sarcoma brain metastasis patients who underwent SRS management, which is within the range reported by other papers, given the relative sample size of patients.

Current SRS local tumor control (LTC) rates of brain metastases from general primary cancers in the literature are reportedly greater than 85%, with a median margin dose range of 18–24 Gy [19,44]. This management approach was supported by Sim et al.’s study of 24 patients with brain metastases from primary sarcoma cancers who underwent SRS management with a median margin dose of 19 Gy (range: 15–24) [43]. Sim et al. reported that prior to SRS management, 20 patients received systemic therapy and 3 patients underwent whole-brain radiotherapy (WBRT) [43]. Additionally, Sim et al. reported that 10 lesions were managed with surgical resection [43]. The authors reported LTC of 89% at 6 months and 89% at 12 months [43]. However, Zamarud reported a lower LTC of 78% at 3 months, 52% at 6 months, and 30% at 12 months when using a median margin dose of 24 Gy in their sarcoma brain metastasis cohort of 23 patients who underwent SRS management [6]. In the present study, we reported LTC of 92.92% based on using a median margin dose of 18 Gy (range: 10–20), which aligns with current values reported in the literature. Our new brain metastasis-free rate was 67.74% after SRS.

Generally, tumor response to SRS among brain metastases has been associated with several factors, including tumor histology [45,46], tumor volume [45,46,47], and extracranial disease control [47]. Among brain metastases from primary cancers, sarcomas typically have a poor prognosis [23,48]. Of sarcoma subtypes reported in the literature, Sim et al. reported a significant association between spindle cell sarcoma and the following variables: local SRS failure (*p* < 0.001) and poor distant tumor control (*p* = 0.003) via univariate analysis [43]. Previous studies have also shown that larger tumor volume is associated with less favorable volumetric response following SRS. Gruber et al. reported that sarcoma and other brain metastases with radioresistant histology and a gross tumor volume > 0.3 cc generated poorer local progression-free survival outcomes (*p* = 0.015) [48]. Likhacheva et al. reported that total tumor volume > 2 cc (*p* < 0.001) and the presence of extracranial disease (*p* < 0.001) were significantly associated with poor OS [49].

The primary AREs of SRS in patients following SRS management of sarcoma brain metastases are tumor necrosis and peritumoral reactive edema. ARE rates reported in the literature for sarcoma brain metastases are not extensively covered but reported at a 1-year cumulative incidence of 13–14% for general brain metastases [50]. Zamarud et al. reported no ARE effects in their cohort of 23 patients who underwent Cyberknife SRS for sarcoma brain metastases [6]. In our study, eight patients developed suspected ARE.

The limitation of this study includes the retrospective and single-institution nature. The dosing delivery protocol in other centers might differ from ours, thus limiting its generalizability. The result in this study reflects a long-term span, during which new systematic agents were developed and would likely have had a significant impact on patients’ outcomes.

## 5. Conclusions

SRS provides a non-invasive and effective approach in sarcoma brain metastasis management. Overall survival is short despite extensive local tumor control. In the future, the use of SRS as an adjuvant may improve survival and quality of life for patients with these rare, aggressive cancers.

## Figures and Tables

**Table 1:** Patient demographics and primary tumor characteristics.

Characteristics	Value
Total no. of pts.	31
Sex	
Male	16 (52%)
Female	15 (48%)
Age at initial primary cancer diagnosis (years)	42.30 ± 21.27 (44 [3–77])
Age at initial brain metastasis diagnosis (years)	46.39 ± 21.57 (47 [3–78])
Time from primary cancer diagnosis to brain metastasis (months)	20 [0–183]
Age at SRS	48.58 ± 21.42 (47 [4–78])
KPS score at SRS	90 [40–100]
Primary histopathology	
Leiomyosarcoma	8 (26%; 7 [87.5%] / 1 [12.5%])
Osteosarcoma	6 (19%; 1 [16.7%] / 5 [83.3%])
Alveolar sarcoma	3 (10%; 1 [33.3%] / 2 [66.7%])
Ewing sarcoma	3 (10%; 2 [66.7%] / 1 [33.3%])
Undifferentiated/unclassified sarcoma	3 (10%; 2 [33.3%] / 1 [66.7%])
Chondrosarcoma	2 (6%; 0 [0%] / 2 [100%])
Other	2 (6%; 1 [50%] / 1 [50%])
Pleomorphic sarcoma	2 (6%; 1 [50%] / 1 [50%])
Liposarcoma	1 (3%; 0 [0%] / 1 [100%])
Rhabdomyosarcoma	1 (3%; 0 [0%] / 1 [100%])
Synovial sarcoma	1 (3%; 1 [100%] / 0 [0%])
Angiosarcoma	0 (0%; 0 [0%] / 0 [0%])
Myxofibrosarcoma	0 (0%; 0 [0%] / 0 [0%])
No. of pts. with concurrent systemic therapy	5 (16%)
Cytotoxic chemotherapy (no. of pts.)	4 (13%)
Molecularly targeted therapy (no. of pts.)	1 (3%)
Immunotherapy (no. of pts.)	0 (0%)
Prior radiotherapy (RT) characteristics	
Type (no. of pts.)	
None	23 (74%)
WBRT	7 (23%)
SRS	1 (3%)
No. of pts. with prior surgical resection	
Partial	6 (19%)
Total/gross	3 (10%)
No. of pts. with systemic disease progression	27 (87%)

No. = number. pts. = patients. KPS = Karnofsky Performance Score. SCC = squamous cell carcinoma. WBRT = whole-brain radiation therapy. F = female. M = male. Values are represented as either number (%), number (%; no. of females [%]/no. males [%]), median [range], or mean ± standard deviation (median [range]).

**Table 2:** SRS planning characteristics.

Characteristics	Value
Metastasis characteristics	
Total No. of sarcoma metastases	113
No. of sarcoma metastases per SRS	2.55 ± 1.80 (2 [1–9])
SRS characteristics	
Margin dose (Gy)	17.06 ± 2.26 (18 [10–20])
Max dose (Gy)	29.51 ± 6.02 (29.45 [19.2–44.4])
Isodose (%)	58.66 ± 12.91 (50 [40–85])
Cumulative tumor volume of sarcoma brain metastases (cc) per pt.	4.20 ± 6.88 (1.4 [0.0041–38.4])
Cumulative V12 volume (cc) per pt.	11.34 ± 10.02 (9.85 [0.0187–40])

No. = number. pt. = patient. Values are represented as either number (%), median [range], or mean ± standard deviation (median [range]).

**Table 3:** Patient outcomes after SRS.

Characteristics	Value
Median overall survival (months) after SRS	7 (0–155)
Local tumor recurrence characteristics	
Recurrence (no. of pts.) at last follow-up	6 (19.3%)
Interval between initial SRS and local tumor recurrence (months) LTC per tumor	3 (0–17) 92.92%
Management of recurrence (no. of pts.)	
SRS	2 (25%)
New brain metastases characteristics	
Progression (# of pts.) at last follow-up	10 (32.26%)
Interval between initial SRS and new brain metastases (months)	6 (1–25)
Management of new brain metastases (no. of pts.)	
SRS WBRT Surgery	12 (92.3%) 1 (7.69%) 1 (7.69%)
No. of pts. with AREs	4 (12.90%)
Median overall survival (months) from brain metastases diagnosis	13.5 (0–156)
Cause of death	
Systemic disease	1 (3.23%)
1-year overall survival (%)	45%
1-year local tumor control (%)	87.10%
1-year new brain metastasis-free control (%)	74.19%

No. = number. pts. = patients. LTC = local tumor control. WBRT = whole-brain radiation therapy. SRS = stereotactic radiosurgery. AREs = adverse radiation effects. Values are represented as either the number (%), median [range], or mean ± standard deviation (median [range]).

**Table 4:** Overall survival univariate analysis.

Variable	Univariate	Multivariate
Age at SRS	0.99 (0.96–1.02), 0.541	-
Type of prior surgery	2.45 (0.45–13.25), 0.297	-
KPS	0.99 (0.94–1.04), 0.624	-
Number of brain metastases	0.72 (0.49–1.05), 0.086	-
Cumulative brain metastasis volume (cc)	1.18 (0.99–1.41), 0.064	-
Presence of extracranial disease	5.18 (1.33–20.11), 0.018 *	-
Concurrent systemic therapy	0.39 (0.07–2.25), 0.294	-
Margin dose (Gy)	0.81 (0.62–1.07), 0.145	-

* Denotes significance (*p* < 0.05).

**Table 5:** Local tumor control univariate analysis.

Variable	Univariate	Multivariate
Sex	0.46 (0.07–2.99), 0.418	-
Age at SRS	1.00 (0.97–1.04), 0.837	-
Type of Prior Surgery	0.47 (0.05–4.37), 0.504	-
KPS	0.99 (0.93–1.05), 0.744	-
Number of brain metastases	1.32 (0.88–1.98), 0.174	-
Cumulative brain metastasis volume (cc)	0.96 (0.85–1.09), 0.568	-
Presence of extracranial disease	4.36 (0.48–39.89), 0.192	-
Concurrent systemic therapy with SRS	1.10 (0.11–11.16), 0.936	-
Prior WBRT	0.74 (0.08–7.15), 0.793	-
Margin Dose (Gy)	1.17 (0.82–1.65), 0.388	-

**Table 6:** Prior sarcoma reports of institutional case series or multi-center studies.

Author/Year	Primary Disease	Management Strategies	# Patients	Median Margin Dose, Gy	Median Time from Primary Dx to Brain Mets. Dx	Median OS from Brain Mets. Dx.	Median OS from SRS to Death	Local Tumor Control	Distant Tumor Control
Flannery et al., 2010 [42]	STS: 5 Leiomyosarcoma: 4 Osteosarcoma: 3 ASPS: 2 Chondrosarcoma: 2 Rhabdomyosarcoma: 1 Ewing’s sarcoma: 1 Neurofibrosarcoma: 1 Liposarcoma: 1 Synovial sarcoma: 1	SRS: 21 Chemotherapy: 14 Resection: 7 WBRT: 5 Supportive care: 3 FRT: 1	21	16	-	16 (range: 1–36) mo.	5 (range: 0.5–30) mo.	4-mo.: 88%	-
Chaigneau et al., 2018 [4]	Other: 82 Leiomyosarcoma: 46 Ewing/primitive neuroectodermal tumor: 30 Liposarcoma: 19 Alveolar soft-part sarcoma: 14 Osteosarcoma: 14 Rhabdomyosarcoma: 14 Angiosarcoma: 14 Synovial sarcoma: 13	WBRT: 144 Systemic therapy: 102 Conservative: 46 Surgery: 38 SRS: 24	246	-	18 (range: 0–215) mo.	2.7 (range: 0–133) mo.	10.2 (5.6–19.9) mo.	-	-
Sim et al., 2020 [43]	Spindle cell sarcoma: 7 Leiomyosarcoma: 4 ASPS: 2 Synovial sarcoma: 2 UPS: 2 Clear cell sarcoma: 1 Ewing sarcoma: 1 Fibroblastic sarcoma: 1 Fibrosarcoma: 1 Liposarcoma: 1 Osteosarcoma: 1 Round cell sarcoma: 1	Systemic therapy: 20 SRS: 24 Surgery: 10 WBRT: 3	24	19 (range: 15–24)	-	6.1 mo.	-	6-mo.: 89% 12-mo.: 89%	6-mo.: 59% 12-mo.: 34%
Zamarud et al., 2023 [6]	HG/HG pleomorphic sarcoma: 4 Spindle cell sarcoma: 4 Undifferentiated sarcoma: 4 Leiomyosarcoma: 2 Synovial sarcoma: 2 Osteosarcoma: 2 Other: 2 Mesenchymal chondrosarcoma: 1 Angiosarcoma: 1 Desmoplastic round cell tumor: 1	SRS: 23 Chemotherapy: 22 Surgery: 15	23	24 (range: 18–30)	-	-	8.2 (range: 0.1–40) mo.	3-mo.: 78% 6-mo.: 52% 12-mo.: 30%	-
Zhang et al., 2024 [23]	Leiomyosarcoma: 10 UPS: 10 Alveolar Rhabdomyosarcoma: 7 Angiosarcoma: 7 Myxofibrosarcoma: 5 Ewing sarcoma: 4 Malignant peripheral nerve sheath tumor: 3 Osteosarcoma: 3 Spindle cell sarcoma: 3 Other: 29	Systemic therapy: 52 WBRT: 39 Surgery: 29 SRS: 24 Other radiation types: 13	81	-	22.8 (range: 0–415.5) mo.	6.0 (range: 0.5–330) mo.	11.6 mo.	-	-
Present Study	Leiomyosarcoma: 8 Osteosarcoma: 6 Alveolar sarcoma: 3 Ewing sarcoma: 3 Undifferentiated/unclassified sarcoma: 3 Chondrosarcoma: 2 Other: 2 Pleomorphic sarcoma: 2 Liposarcoma: 1 Rhabdomyosarcoma: 1 Synovial sarcoma: 1	SRS: 31 Surgery: 9 WBRT: 7 Systemic therapy: 5	31	18 (range: 10–20)	20 (range: 0–183) mo.	13.5 (range: 0–156)	7 (range: 0–155) mo.	1-yr: 87.1%	1-yr: 74.19%

Brain Mets. = brain metastases. Dx = diagnosis. SRS = stereotactic radiosurgery. WBRT = whole-brain radiotherapy. mo. = month. ASPS = alveolar soft part sarcoma. STS = soft-tissue sarcoma. UPS = undifferentiated pleomorphic sarcoma. PNET = peripheral neuroectodermal tumor.

## Data Availability

Data are contained within the article.

## Abbreviations

The following abbreviations are used in this manuscript:

SRS	Stereotactic radiosurgery
AREs	Adverse radiation effects
OS	Overall survival
LTC	Local tumor control
KPS	Karnofsky performance score
